# High-Content Analysis of Sequential Events during the Early Phase of Influenza A Virus Infection

**DOI:** 10.1371/journal.pone.0068450

**Published:** 2013-07-12

**Authors:** Indranil Banerjee, Yohei Yamauchi, Ari Helenius, Peter Horvath

**Affiliations:** 1 Institute of Biochemistry, ETH Zürich, Zurich, Switzerland; 2 Light Microscopy and Screening Centre, ETH Zürich, Zurich, Switzerland; University of Edinburgh, United Kingdom

## Abstract

Influenza A virus (IAV) represents a worldwide threat to public health by causing severe morbidity and mortality every year. Due to high mutation rate, new strains of IAV emerge frequently. These IAVs are often drug-resistant and require vaccine reformulation. A promising approach to circumvent this problem is to target host cell determinants crucial for IAV infection, but dispensable for the cell. Several RNAi-based screens have identified about one thousand cellular factors that promote IAV infection. However, systematic analyses to determine their specific functions are lacking. To address this issue, we developed quantitative, imaging-based assays to dissect seven consecutive steps in the early phases of IAV infection in tissue culture cells. The entry steps for which we developed the assays were: virus binding to the cell membrane, endocytosis, exposure to low pH in endocytic vacuoles, acid-activated fusion of viral envelope with the vacuolar membrane, nucleocapsid uncoating in the cytosol, nuclear import of viral ribonucleoproteins, and expression of the viral nucleoprotein. We adapted the assays to automated microscopy and optimized them for high-content screening. To quantify the image data, we performed both single and multi-parametric analyses, in combination with machine learning. By time-course experiments, we determined the optimal time points for each assay. Our quality control experiments showed that the assays were sufficiently robust for high-content analysis. The methods we describe in this study provide a powerful high-throughput platform to understand the host cell processes, which can eventually lead to the discovery of novel anti-pathogen strategies.

## Introduction

In the field of infectious diseases, the use of high-content perturbation screens using siRNAs, shRNAs, and chemical agents is rapidly expanding. Information regarding cellular factors that assist viruses and other intracellular pathogens during replication in the host cell, and on pharmacological agents that affect infection is increasing. To understand disease mechanisms, and to develop novel antiviral strategies, it is important to precisely define the event in the viral replication cycle that is affected. Knowing the identity of a gene that promotes/inhibits infection, or a drug that blocks infection is not sufficient. Since the number of ‘hits’ provided by genome-wide and drug screens is generally large, such a method must be high-throughput. In this study, we describe a series of such assays for early events of influenza A virus (IAV) infection in tissue culture cells.

**Figure 1:**
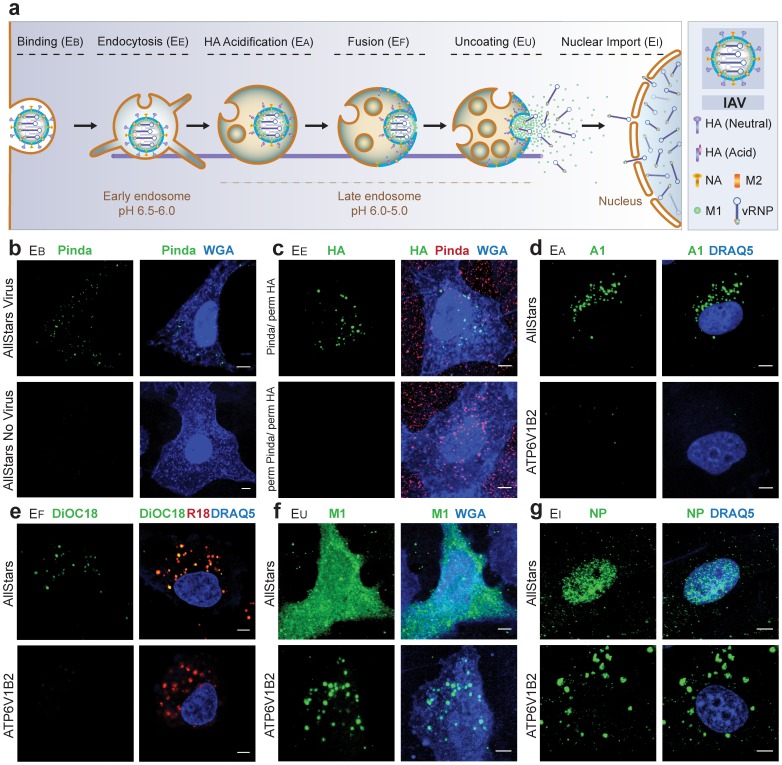
Sequential events during host-cell entry of IAV. (a). Entry involves six steps; binding of the virus to the cell membrane (EB), internalization by endocytosis (EE), acidification in late endocytic vacuoles (EA), fusion of viral and vacuolar membranes (EF), uncoating of nucleocapsid (EU), and nuclear import of vRNPs (EI). Components of IAV are shown in the right (NA: neuraminidase, M2: proton channel). (b–g). High-resolution confocal images of the individual assays. (b) Binding (EB assay): *(Top)* AllStars negative siRNA-treated cells were incubated with IAV for 1 h in the cold. After washing, cell-bound virus particles were stained by IIF using the Pinda antibody against HA (green). The cells membrane was visualized with WGA-AF647 (blue). (*Bottom*) Cells with no virus (c) Endocytosis (EE assay): *(Top)* Cells were incubated with IAV for 1 h in the cold. After washing, cells with bound viruses were warmed up to 37°C for 20 min to allow virus internalization. To distinguish between the endocytosed and extracellular virus particles, the HA epitopes of the virus particles accessible from the medium were masked with the Pinda antibody. The cells were then permeabilized with detergent and incubated with a mouse monoclonal antibody (HA1). After fluorescently-labeled secondary antibody treatment, the endocytosed (green) and non-internalized virus particles (red) were identified (Pinda/perm HA). Cell membrane (blue) was stained with WGA. (*Bottom*) After virus internalization and fixation, cells were permeabilized with detergent and similar staining procedures were followed. The endocytosed and extracellular virus particles are not distinguished and both showed same fluorescent signal (red) (perm Pinda/perm HA). (d) Acidification (EA assay): *(Top)* Virus particles were allowed to enter the AllStars negative siRNA-treated cells at 37°C for 1.0 h and were stained with A1 antibody to detect the acid-induced conformation of HA (green) in endocytic vacuoles near the nucleus (blue). (*Bottom*) Cells treated with ATP6V1B2 siRNA showed no A1 signal due to block in endosome acidification. (e) Fusion (EF assay): *(Top)* Virus particles were labeled with SP-DiOC18 (3) and R18, and were allowed to enter the AllStars negative siRNA-treated cells at 37°C for 1.5 h, after which the cells were fixed. Fusion of viral and vacuolar membranes of cells triggered dequenching of DiOC18(3) (green). DiOC18(3) signal colocalized with the R18 (red) signal. (*Bottom*) Cells treated with ATP6V1B2 siRNA showed R18 (red) signal only. (f) Uncoating (EU assay): *(Top)* To detect the dispersal of M1 into the cytoplasm of the cells (blue), viruses were allowed to enter the AllStars negative siRNA treated cells at 37°C for 3 h. After fixation and permeabilization, mouse monoclonal antibody HB64 was used to stain the viral M1 (green). (*Bottom*) Block in uncoating due to ATP6V1B2 siRNA treatment, where the virus particles (green) accumulated in the endocytic vacuoles. (g) Nuclear import (EI assay): *(Top)* In the AllStars negative siRNA-treated cells, virus particles were allowed to enter at 37°C for 3.5 h. Incoming NP proteins (green) were detected within the nucleus (blue) by the treatment with mouse monoclonal antibody HB65. (*Bottom*) Import of NP (green) was blocked in cells treated with ATP6V1B2 siRNA. *Scale bar = 5* *µm.*

IAVs are enveloped viruses belonging to the family *Orthomyxoviridae* with a negative-stranded, segmented RNA genome. To deliver their genome in the form of 8 viral ribonucleoproteins (vRNPs) into host cells, IAVs take advantage of the endocytic and cytosolic trafficking machinery of the host. After binding to sialic acid-containing receptors on the plasma membrane, IAV particles are internalized by clathrin-mediated endocytosis and macropinocytosis [1], [2]. After sorting to late endosomes or mature macropinosomes, they are exposed to low pH (5.5–5.0), which induces an irreversible conformational change in the viral hemagglutinin (HA, an envelope glycoprotein), activating its membrane fusion activity [3]. The viral envelope fuses with the limiting membrane of the endosome, and the capsid is released into the cytoplasm. The matrix protein M1 and the vRNPs dissociate from each other. The vRNPs are imported into the nucleus for transcription and replication of viral genes [4], whereas the M1 disperses into the cytosol (Figure 1a).

High rates of mutation and the possibility of re-assortment facilitate generation of new IAV strains, decreasing the effect of vaccines and drugs. Therefore, instead of targeting the virus itself, it may be advantageous to develop antiviral strategies that interfere with host cell factors essential for viral entry and replication. For this, systematic identification of processes that promote viral infection is necessary. Recently, five genome-wide RNAi screens for IAV infection were performed in tissue culture cells. Collectively, about 1000 genes were identified as factors that support the IAV replication cycle [5]. However, the precise role of most of these factors at different stages of the viral life cycle was not elucidated. Therefore, development of assays for the sequential steps in the infectious cycle is warranted to functionally classify hits according to the step in the entry program affected, and this in a high-throughput manner.

We developed image-based assays to quantify seven steps in the early stages of the replication cycle as depicted in Figure 1a. These were: 1) Virus binding to the cell membrane (for brevity, we call the assay for binding the EB assay), 2) Endocytic uptake of incoming virus (the EE assay), 3) Acidification of HA in late endosomes (the EA assay), 4) Fusion of viral and endosomal membranes (the EF assay), 5) Nucleocapsid uncoating in the cytosol (the EU assay), 6) Nuclear import of vRNPs (the EI assay), and 7) Expression of the nucleoprotein (NP), an early viral product. To quantify the information, we developed protocols for image and data analysis. In some of the assays, we used single parametric analysis. For others, where cellular phenotypes were detectable, but could not be precisely described by a single parameter, we implemented supervised machine learning.

The assays made it possible to determine the kinetics of crucial steps during cell entry of IAV, and to define ‘time-windows’ when each event could be optimally detected. When coupled with high-content analysis, the techniques can be used to address biological questions related to IAV entry, and to identify key functions provided by host factors during the early stages.

## Results and Discussion

The influenza A virus strain used in this study was an H3N2 strain called X31, which is a high-growth reassorted strain derived from the A/Puerto Rico/8/34 (PR8) and A/Hong Kong/1/68 strains. The cells were A549 cells (a human alveolar epithelial cancer cell line). To be able to monitor the passage of incoming viruses through steps of the entry program, we developed fluorescence microscopy-based methods. We will first discuss the approaches taken to visualize the viruses in different phases of entry, and then describe how the data was used to design quantitative high-throughput assays.

### Detection of IAV Binding to Cell Membrane (EB Assay) and Viral Endocytosis (EE Assay)

To detect binding of viruses to cells (EB assay), we incubated purified IAV with cells at 4°C for 1 h. The cells had been transfected with scrambled control siRNAs called AllStars, which we used as a negative control throughout the study. Indirect immunofluorescence (IIF) using a rabbit polyclonal antibody (Pinda) against HA [6] was used to label the viruses (green), and a fluorescent marker (wheat germ agglutinin, WGA) to define the location of cells (blue) (Figure 1b, Table S1a). By confocal microscopy, the viruses could be visualized as spots distributed over the cells. In a control experiment, we treated the cells with neuraminidase prior to the EB assay. Neuraminidase hydrolyzes the glycosidic linkages between cellular surface glycoproteins and sialic acids, the latter being attachment factor for IAV. We observed almost no binding of IAV particles to the cell membrane of neuraminidase-treated cells, whereas viral binding was normal in the mock-treated cells (Figure S1).

To detect endocytosis (EE assay), cells with bound viruses were warmed up to 37°C for 20 min and then fixed with 4% formaldehyde. To distinguish between particles in the cytoplasm from virions still on the cell surface, we first masked the HA epitopes of particles accessible from the medium with the Pinda antibody. After a second fixation, the cells were permeabilized with detergent and incubated with a mouse monoclonal antibody against HA, called HA1 [7]. After staining with appropriate fluorescently-labeled secondary antibodies, the endocytosed and non-internalized virus particles could be distinguished by confocal microscopy (we call this staining procedure ‘Pinda/perm HA’) (Figure 1c, Table S1b). External viruses were red and internalized particles were green. As previously reported [8], the internalized particles were present in brightly fluorescent spots mainly in the perinuclear region of the cell. If the cells were not allowed to internalize viruses by keeping them on ice, no virus particles were detected with the HA1 antibody following the Pinda antibody treatment (data not shown). If the cells were permeabilized before the treatment with Pinda antibody, no staining with the HA1 antibody was seen. This indicated that the Pinda antibody masked the HA epitopes sufficiently (perm Pinda/perm HA) (Fig1c, bottom). When the cells were not permeabilized at all during staining (Pinda/HA), only the non-internalized virus particles were detected.

It was observed that in the non-permeabilized cells (Pinda/HA), WGA stained both the cell membrane and the nucleus after fixation, resembling the WGA staining pattern of the permeabilized cells. This indicated that the fixation procedure allowed WGA to access the cytoplasm of the cells. However, the HA1 antibody did not stain viral HA, the ectodomain of which is located in the lumen of endosomes. This observation demonstrated that the EE assay distinguished the endocytosed versus non-internalized virus particles.

### Detection of the Acid-induced Conversion of HA (EA Assay) and Viral Membrane Fusion (EF Assay)

When IAV is exposed to a pH below 5.5, the HA undergoes an irreversible conformational change that can be detected using a monoclonal antibody A1 (EA assay) [9]. When cells with internalized viruses were subjected to IIF using the A1 antibody, the labeled HA was, as expected, localized exclusively in the perinuclear vacuoles (Figure 1d, Table S1c). Conversion of HA to the acid-induced conformation was inhibited by using siRNA-based depletion of ATP6V1B2, a subunit of the vacuolar-ATPase (vATPase) required for endosome acidification (Figure 1d, bottom). Western blotting of AllStars and ATP6V1B2 siRNA-treated cells showed significant decrease of ATP6V1B2 protein expression in the cells treated with ATP6V1B2 siRNA (Figure S2).

To monitor fusion between the IAV envelope and cellular membranes (EF assay), we used a lipophilic dye-based fluorescence dequenching assay using R18 (red) and SP-DiOC18 (green, fixable). In the labeled virus, the green fluorescence is suppressed by both self-quenching of DiOC18 and fluorescent resonance energy transfer (FRET) from DiOC18 to R18, whereas the red fluorescence from R18 is partly self-quenched [10]. Labeled virus was allowed to enter cells for 1.5 h, and then fixed. When viewed by confocal microscopy, the cells showed numerous perinuclear vacuoles with both red and green fluorescence. This indicated that viral fusion had occurred (Figure 1e, Table S1d). Cells in which acidification of endosomes was inhibited following depletion of ATP6V1B2, only R18 fluorescence was detected, indicating that fusion did not take place (Figure 1e, bottom).

### Detection of Nucleocapsid Uncoating (EU assay), Nuclear Import of vRNPs (EI Assay), and NP Translation

The acid-activated fusion of IAV envelope with the limiting membrane of endocytic vacuoles results in the transfer of the capsid into the cytosol. We monitored uncoating of the capsid by following the M1 protein, the major component of the capsid (the EU assay). When the capsids undergo dissociation, M1 disperses into the cytosol and becomes more accessible to a monoclonal antibody (HB64) [11]. Thus, uncoating resulted in M1 redistribution throughout the cytoplasm and a dramatic increase in signal intensity (Figure 1f, Table S1e). In control cells with the vATPase inhibited, no uncoating was detected (Figure 1f, bottom).

The vRNPs liberated through the uncoating process have nuclear import signals, and are imported into the nucleoplasm. In the EI assay, we followed the nuclear accumulation of vRNPs (11). They could be visualized by IIF using a monoclonal antibody (HB65) against NP, the major protein component of vRNPs (Figure 1g, Table S1f). The vRNPs failed to accumulate in the nucleus in the cells in which ATP6V1B2 was depleted (Figure 1g, bottom).

Finally, the synthesis of NP in cells infected at low multiplicities of infection in the absence of cycloheximide was used as an indication of early viral protein transcription and synthesis (Figure S3, Table S1g). This was our assay for infection.

### Image Acquisition and Data Quantification

For automated, high-throughput analysis, we optimized the procedures for the 96-well-plate format and automated microscopy using a 20× objective, and developed robust quantification methods. Typical images acquired with automated microscopy are shown in Figure S4. All the results were based on at least three experiments performed on separate days.

**Figure 2:**
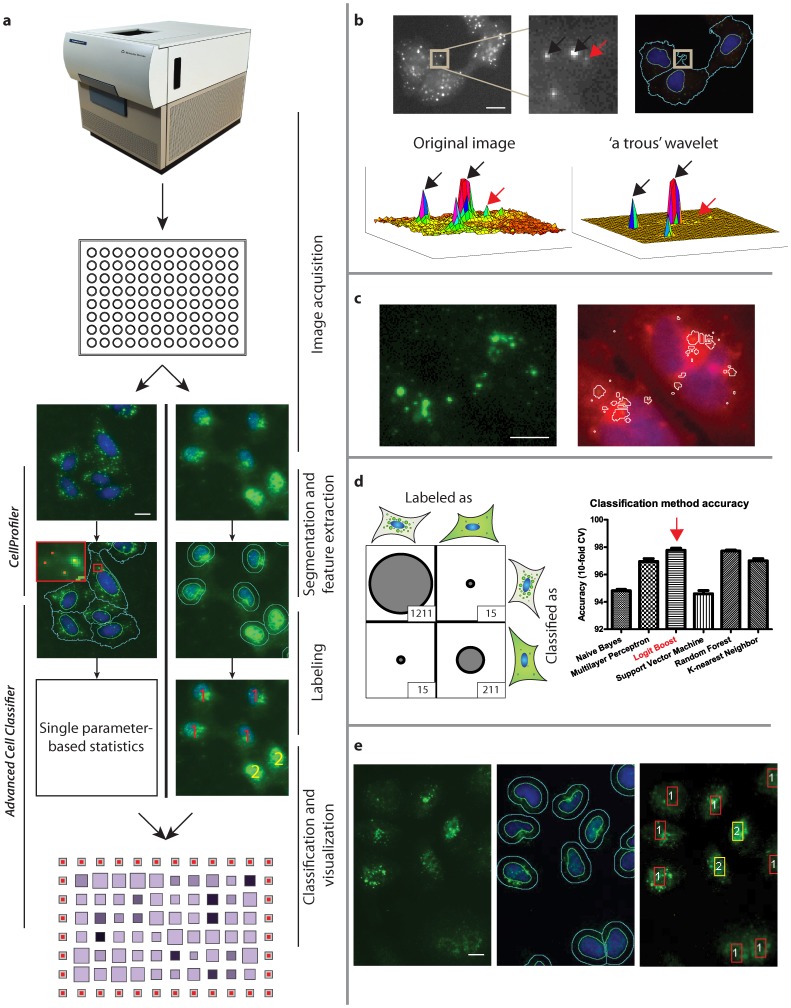
Data analysis steps. (a) Data analysis pipeline. Alternative concepts: *(left column)* single parameter-based statistics, *(right column)* machine learning. (b) Spot detection for the EE assay. Spot intensities with the desired size *(black arrows)* were amplified, while noise *(red arrow)* and uneven background was suppressed. (c) Analysis of the EA and EF assays. GFP intensity was thresholded, and the detected objects were filtered by size. (d) (*Left)* EU assay confusion matrix. (*Right)* Comparison of classification methods using 10-fold cross validation. *Logistic regression classifiers with boosting* (LogitBoost) were the most accurate (∼98%) *(red arrow)*. (e) EI assay (*Left)* Original image. (*Middle)* Segmentation result. (*Right)* Phenotypic classification of cells: [1] import-negative [2] import-positive.

To quantify the data, we used two approaches. The first was to extract and analyze a single parameter to describe the biological phenomenon (Figure 2a, left). The second and more novel, was to extract multiple (many dozens to hundreds) of parameters per cell and to use machine learning [12], [13] to reduce complexity (Figure 2a, right).

**Figure 3:**
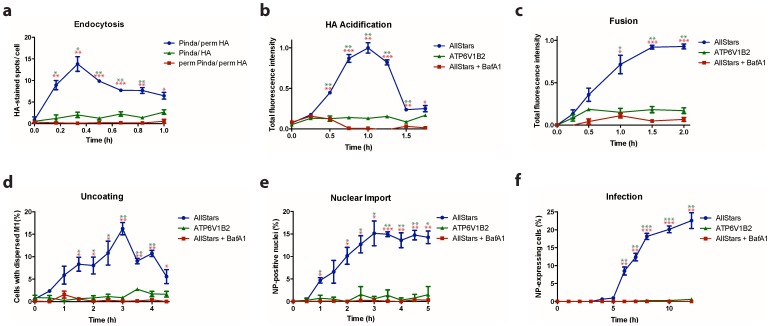
Time-course of IAV entry as shown by individual assays. (a) Kinetics of IAV endocytosis in the ‘Pinda/perm HA’, ‘Pinda/HA’ and ‘perm Pinda/perm HA’ cells. Endocytosed IAV signal in the ‘Pinda/perm HA’cells peaks at 20 min post-infection. (b) Acidification time-course in the cells treated with AllStars negative and ATP6V1B2 siRNAs, and the cells treated with 50 nM Bafilomycin A1 (BafA1) to block endosomal acidification. The acidification signal in the AllStars negative siRNA-treated cells reaches the peak at 1 h post-infection. (c) Kinetics of viral fusion, which shows the dequenching signal from DiOC18(3) in the AllStars negative siRNA-treated cells peaks at 1.5 h post-infection. (d) Nucleocapsid uncoating time-course indicating the peak of M1 dispersal signal is at 3 h post-infection. (e) Nuclear import time course shows that the import plateaus at 3.5 h post-infection in the control cells. (f) Kinetics of infection (transcription and translation of NP), which shows that the optimal time for the detection of cells with newly synthesized NP is 8 h post-infection. Z’ factor values are represented by * - between 0 and 0.5; ** - between 0.5 and 0.8; and ***>0.8.

The single parameter approach was used for virus binding (EB assay), endocytosis (EE assay), HA acidification (EA assay), and fusion (EF assay). This was because in these assays, the signal was homogenous, and the phenotypes were distinct. For the post-fusion assays i.e. the uncoating (EU assay), nuclear import (EI assay), and the NP translation assay, the signal was more heterogeneous and non-synchronous. This was most likely due to the increased involvement of cytoplasmic cellular factors in these processes. Therefore, for quantification we chose the second method and utilized all available cellular features. We initially tested a single parameter method (spot detection) for the E_I_ assay. However, the reliability was low as shown by the low Z’ factor [14] scores between ATP6V1B2-depleted and AllStars negative controls. (Figure 3e and Figure S5c, d).

After image acquisition, individual cell nuclei, cell borders, and virus particles were segmented. For the EB, EE, EA, and EU assays, the exact cell shape was determined. For the EI assay, a 12-pixel-wide ring around the nucleus was used to represent the cytoplasm (Figure 2e). In the EE assay, the number of virus-containing endosomal vacuoles was detected using ‘a trous’ wavelet transform [15] (Figure 2b). For the EA assay, the integrated intensity of A1 staining was determined (Figure 2c). For the EU and EI assays, the texture, intensity, and morphological features were extracted based on the segmented regions. These features were later used for machine learning. Image analysis was performed with a customized version of the CellProfiler program [16]. Details of the analysis can be found in the Materials and Methods section and Table S2.

For the EU, EI and NP translation assays, supervised machine learning was used. This method is useful for quantifying samples whose features cannot be described with a single feature (Figure 2d, e). Since it was not known which of the different machine learning methods was most optimal for single cell-based (SCB) analysis, we tested the most conventional methods and found that the logistic regression classifiers with boosting gave the most reliable results (Figure 2d, Figure S6). For SCB labeling and classification, we used the Advanced Cell Classifier program [12] (Figure S7), which incorporates learning algorithms from the WEKA package [17]. The phenotype recognition accuracy was above 95% for the EU and EI assays. A description of the analyzed classification methods is provided in Materials and Methods. A list of extracted features, the custom CellProfiler modules, and the pipelines for image analysis of the individual assays can be found on the website www.highcontentanalysis.org.

### Defining Optimal Time Points for Detection

To determine the optimal time points for each of the six IAV entry assays and the NP translation assay, detailed time course experiments were performed. As shown in Figure 3, the time point at which the respective signal peaked or plateaued followed the expected order of IAV entry steps: endocytosis at 20 min; HA acidification at 1 h; fusion at 1.5 h; uncoating at 3 h; nuclear import at 3.5 h; and NP synthesis at 8 h (Figure 3). This is to our knowledge the first time that time course of steps during IAV entry has been analyzed in any detail. While each step showed non synchrony, the apparent half times suggested a 12 min lag between endocytosis and HA acidification, a 15 min lag between acidification and fusion, a 45 min lag between fusion and uncoating, and a further 30 min lag between uncoating and vRNP import into the nucleus.

Based on the time-course experiments, optimal time points for the high-throughput assays were defined. The reduction in the signal following the peaks in the EE, EA, and EU assays was probably due to modification or degradation of the respective viral antigens (Figure 3a, b, and d). Depletion of ATP6V1B2 blocked HA acidification and subsequent processes, but binding of virus to the cell membrane remained unperturbed (Figure S8).

The synthesis of NP was used as a read-out for IAV infection (Figure 3f). Other methods to detect influenza virus infection have been used for high-throughput analysis, such as detecting the surface expression level of HA [18]. In another study, a reporter virus was generated that encoded *Renilla* luciferase [19], and luciferase activity at different time points post-infection served as an indicator of viral replication. To infect Drosophila DL1 cells, a modified influenza virus was generated in which the HA was replaced with the glycoprotein of vesicular stomatitis virus (VSV-G), and the neuraminidase gene with *Renilla* luciferase [20].

To evaluate our high-throughput platform, we tested cellular factors known to mediate steps in IAV entry. IAV uses clathrin-mediated endocytosis as one of its endocytic mechanisms [1], and the GTPase dynamin is required for pinching off the newly-formed vesicles. We found that a pharmacological inhibitor of dynamin, dynasore, blocked IAV endocytosis by 80% at 20 min (Figure S9). When we knocked down two additional components of the vATPase other than ATP6V1B2, namely ATP6AP2 and ATP6V1A, HA acidification was significantly reduced (Figure S9). Cullin-3 (CUL3), a scaffolding subunit in a large family of E3 ubiquitin ligases, is involved in late endosome maturation and promotes IAV capsid uncoating [21]. We confirmed that in CUL3-depleted cells uncoating was blocked. CSE1L depletion inhibited vRNP import as shown previously [8] (Figure S9). All these results confirmed the reliability of our assays.

To further assess the robustness of our analyses, the Z’ factor [14] was determined for every time-point in all the assays (Figure 3). The z’ factor was higher than 0.5 for all the peak/plateau time-points. This indicated the robustness of the readout values and an excellent separation between positive and negative controls.

### Conclusions

Through image processing programs, it was possible to computationally analyze and quantify the effects of perturbations with high confidence in all major steps of IAV entry, and in NP synthesis (Figure 3). The advantage of the single parameter approach is that one can interpret the results intuitively. In contrast, machine learning does not require additional analysis steps by a computer vision expert, and the decision-making process is based solely on the expertise of the biologist. Our assay systems are sufficient to analyze the IAV entry pathway. In a modified form, they can be easily applied to other viruses and intracellular pathogens. They provide a platform to promote the understanding of dynamic biological processes through high-content screening and will contribute to the discovery of anti-viral strategies that target host cell factors.

## Materials and Methods

### Cell Culture and Virus Preparation

A549 ATCC cells were cultured in Dulbecco's modified Eagle's medium (D-MEM) (Invitrogen), supplemented with 10% FCS, 1% GlutaMAX and 1% non-essential amino acids. The cells were grown as monolayers and passaged biweekly. Influenza A X31 strain (an H3N2 reassorted strain derived from the A/Puerto Rico/8/34 (PR8) and A/Hong Kong/1/68 strains) was purchased from Virapur (CA, USA) in purified form. To propagate the influenza virus, 60 pathogen-free chicken eggs were inoculated with the virus and incubated at 33–37°C for 2 days. The allantoic fluid was harvested and clarified by low-speed centrifugation, which was then concentrated by high-speed centrifugation. To further concentrate the virus, two rounds of 10–40% sucrose gradient centrifugation were carried out. Viral bands were harvested, pooled and re-suspended in formulation buffer (40% sucrose, 0.02% BSA, 20 mM HEPES pH 7.4, 100 mM NaCl, 2 mM MgCl_2_). The viral titer was determined (2.4×10^5^ TCID_50_ infectious units/µl) in MDCK cells. The virus was aliquoted and stored at −80°C until use.

### siRNA Transfection

siRNAs (AllStars, ATP6V1B2, ATP6AP2, ATP6V1A, CUL3, and CSE1L) were purchased from QIAGEN and reverse-transfection was carried out with a final concentration 10 nM onto A549 cells in 24-well plates containing coverslips or 96-well optical-bottom Matrix plates (Thermo Scientific). The sequences of the above siRNAs are enlisted in the Table S3. Lipofectamine RNAiMax (Invitrogen) and D-MEM were mixed at a ratio 1∶150. siRNAs were added, gently mixed, and incubated at room temperature (RT) for 1 h. Cells were trypsinized, counted and plated directly onto the siRNA-lipofectamine complex mixture. The number of cells plated in each well of the 24-well and 96-well plates was 12500 and 1500, respectively. Following transfection, the cells were kept in a 5% CO2 incubator at 37°C for 72 h, after which the entry assays were performed.

### Antibodies and Reagents

Anti-X31 rabbit polyclonal antibody (Pinda) and anti-HA monoclonal antibody (A1) specific for the post-acid conformation of HA have been previously described [6], [7]. Hybridoma cell lines producing monoclonal antibody against IAV matrix protein (HB64), and nucleoprotein (HB65) were purchased from ATCC. Anti-ATP6V1B2 and anti-β actin antibodies were purchased from LifeSpan Biosciences and Sigma-Aldrich, respectively. R18 and SP-DiOC18(3) (Invitrogen) were re-suspended in EtOH and used at a final concentration of 0.4 µM and 0.2 µM, respectively. Labeling was performed as previously described [10]. DRAQ5 was purchased from Biostatus Limited. Hoechst 33258 and wheat germ agglutinin (WGA) were purchased from Invitrogen. Bafilomycin A1 (BafA1), cycloheximide, and formaldehyde solution (36%) were from Sigma-Aldrich.

### Western Blot

A549 cells were transfected with 10 nM siRNA for 3 days. Cells were then harvested and subjected to Western blotting.

Detection of the ATP6V1B2 and β actin proteins was done with anti-ATP6V1B2 (1∶1000 dilution) and anti-β actin (1∶3000) antibodies, respectively.

### IAV Entry Assays

Indirect immunofluorescence techniques were used to detect viral components at different steps of IAV entry. Both confocal laser-scanning and automated high-content fluorescence microscopy were used to acquire the images as described below:

### A. Virus Internalization Conditions

IAV diluted in infection medium (D-MEM, 50 mM HEPES pH 6.8, 0.2% BSA) at 4°C for 1 h were allowed to bind to siRNA-transfected A549 cells on ice. The cells were then washed with ice-cold infection medium to remove the unbound virus particles. The bound particles were allowed to internalize at 37°C in a CO_2_ incubator for different time periods. To prevent the synthesis of new viral proteins in the EE, EA, EF, EU, and EI assays 1 mM cycloheximide was included in the infection medium during IAV binding and internalization. In the EU and EI assays, fresh infection medium containing cycloheximide was exchanged every 2 h post-internalization to ensure optimal efficacy of the drug. For the control of the EA, EF, EU, EI and infection assays, cells were either transfected with siRNA ATP6V1B2 targeting a vATPase subunit, or treated with 50 nM BafA1 during internalization. In both the control samples, acid-exposure of IAV in late endosomes was prevented, and as a consequence of which HA activation and down-stream processes in entry were blocked. The virus amounts used from the stock (2.4×10^5^ TCID_50_ infectious units/µl) in each well of a 24-well or 96-well plate and the detection time point for each assay are summarized in Table S1.

### B. Entry Assay Techniques

#### 1. Binding (EB assay)

siRNA-transfected A549 cells were incubated with IAV in infection medium at 4°C for 1 h. After washing 3 times with ice-cold PBS, the cells were fixed with 4% formaldehyde at RT, rewashed and stained with WGA-AF647 in PBS (1∶250 dilution) for 30 min at RT. After a further washing step, the cells were incubated with Pinda antibody (1∶10000) in blocking solution (BS) (1% BSA, 5% FCS in PBS) for 1 h at RT, washed with PBS, and stained with secondary anti-rabbit IgG-AF488 conjugate in BS (1∶1000) together with Hoechst 33258 (1∶10000) for 1 h at RT.

#### 2. Endocytosis (EE assay)

siRNA-transfected cells were incubated with virus in the cold as in B1, and the bound virus allowed to be internalized for different time periods as describe above. After washing, they were fixed, washed with PBS, and the cell membrane was stained with WGA-AF647 in PBS (1∶250) for 30 min at RT and washed again to remove unbound WGA-AF647. The epitopes of extracellular HA were blocked overnight at 4°C with Pinda antibody (1∶500) in BS, and the cells stained with secondary anti-rabbit IgG-AF594 conjugate in BS (1∶1000) for 1 h at RT. After fixation in 4% formaldehyde for 20 min and washing, a permeabilization solution (PS) (0.1% saponin, 1% BSA, 5% FCS in PBS) was added for 30 min followed by incubation with a mouse monoclonal antibody specific for HA1 in PS (1∶100) for 2 h at RT. After washing, the cells were incubated with secondary anti-mouse IgG-AF488 (1∶1000) in PS for 1 h, and the nuclei were stained by Hoechst 33258 (1∶10000) for high-content automated microscopy. This method (referred to as Pinda/perm HA) efficiently distinguishes between the endocytosed and the non-internalized particles. In control samples, the antibody staining was done exclusively either in PS (perm Pinda/perm HA) or in BS (Pinda/HA). In cells following the perm Pinda/perm HA procedure, the endocytosed virus particles could not be distinguished from the non-internalized particles. In Pinda/HA cells, only the non-internalized particles were detected.

#### 3. Acidification (EA assay)

The cells were permeabilized with PS for 30 min at RT. The cells were then incubated with mouse monoclonal A1 antibody in PS (1∶1000) for 2 h, washed with PBS, and incubated with secondary anti-mouse IgG-AF488 (1∶1000) in PS for 1 h together with either DRAQ5 (1∶1000) or Hoechst 33258 (1∶10000) in PS.

#### 4. Fusion (EF assay)

IAV stocks were diluted in PBS to 0.1 mg/ml and labeled for 1 h at RT with R18 and SP-DiOC18 (3) at final concentrations of 0.4 µM and 0.2 µM, respectively. The labeled virus particles were filtered through a 0.22 µM-pore filter (Millipore) and stored at 4°C in the dark till use. After internalization and fixation, nuclei were stained with either DRAQ5 (1∶1000) or Hoechst 33258 (1∶10000) in BS.

#### 5. Uncoating (EU assay)

The cell membrane was stained with WGA-AF647 as described above. The cells were permeabilized with PS for 30 min at RT, and incubated with purified mouse monoclonal antibody HB64 in PS (1∶250) for 2 h to stain the viral M1. The cells were washed with PBS, followed by incubation with secondary anti-mouse IgG-AF488 (1∶1000). Nuclei were stained with Hoechst 33258 (1∶10000).

#### 6. Nuclear import (EI assay)

The cells were permeabilized with PS for 30 min at RT, and incubated with mouse monoclonal antibody HB65 (hybridoma supernatant) in PS (1∶10) for 2 h to stain the incoming viral NP. The cells were washed with PBS, followed by incubation with secondary anti-mouse IgG-AF488 (1∶1000). Nuclei were stained with either DRAQ5 (1∶1000) or Hoechst 33258 (1∶10000).

#### 7. Infection

Newly synthesized NP was detected as described in **6**.

### Image Acquisition

For high-resolution imaging, specimen on coverslips from 24-well plates were mounted on a glass slide with Immu-mount (Thermo Scientific) and viewed on a Zeiss LSM 510 laser scanning confocal microscope. Both 100× and 63× objectives (1.4 numerical aperture and 1×1 binning) were used to acquire images. Automated image acquisition of 96-well Matrix plates was performed with a 20× objective (0.75 numerical aperture and 1×1 binning) using Molecular Devices ImageXpress Micro imaging system. From each well, 9 images (3×3) were acquired for each channel.

### Image Analysis

Image analysis steps were performed using the CellProfiler program [16]. The analysis of all screens involved five major steps: *(1)* Image intensities were converted from standard microscopic format (tiff, 12 bit) to real values. *(2)* Cell nuclei and cytoplasm were identified. These segmentation steps thresholded the image using adaptive methods and cells touching each other were split using watershed method. *(3)* Identification of subcellular structures. In case of the EE assay, a spot detection algorithm was implemented based on ‘a trous’ wavelet transform, to amplify the signal of spots in a given size and to suppress noise, background instabilities, and objects out of the size range [15]. *(4)* For the EU and EI assays, intensity, morphological, and textural cellular properties were extracted. *(5)* Refactoring of the analysis data. For the EE assay, the output was the number of virus containing particles per cell. For the EB, EA and EF assays, the integrated viral intensity per cell was extracted. For the EF assay, the mean background green fluorescence value of time point zero was subtracted from all the measurements. For the EU, EI, and the infection assays, the output consisted of 27–48 features per cell. Table S2 contains the detailed list of performed steps for each assay. The image analysis calculations were done on a high-performance cluster machine. The usual runtime of the calculation was ∼1 minute/site/node. (e.g. a 96-well plate, 9 sites/well, running 32 parallel jobs takes 27 min). The CellProfiler pipelines, the custom modules, the refactoring functions, and a detailed list of features can be downloaded in www.highcontentanalysis.org.

### Multi-parametric Phenotype Classification

For the EU, EI, and the NP translation assays, single cell-based (SCB) phenotypic profiling was used based on multi-parametric analysis. For this purpose, we used the Advanced Cell Classifier program [12] (www.cellclassifier.org), which allows the user to assign predefined phenotypes to cells. The computer uses this training set to learn a model and to classify unassigned cells through several machine learning methods (Figure S5). To find the best method, we compared the 10-fold cross validation accuracy of the most commonly used classification methods i.e. Multilayer Perceptron ( = Artificial Neural Networks), Logit Boost ( = logistic regression with boosting), Support Vector Machine, Random Forest, and K-nearest Neighbor. Logit Boost with minor improvements was the most optimal method for all of the assays. We also tested the Naive Bayesian method and found that using advanced methods significantly increased accuracy [12] (Figure 2d, Figure S6a). The WEKA implementation of the machine learning methods was used with default parameters [17]. In Figure S6b we show the receiver operating characteristics (ROC) curves [22] for the EI assay. Both the cross validation and ROC analysis show high recognition rates (CV >95% and AUC >0.99), making the analysis robust.

## Supporting Information

Figure S1 **IAV binding in the neuraminidase and mock-treated cells.** A549 cells were treated with 0.25 units/ml neuraminidase at 37°C for 4 h, followed by EB assay. Images were acquired with a confocal microscope. The HA of IAV was stained with Pinda antibody (green), and the cell membrane was stained with WGA (blue).
(TIF)

Figure S2 **Western blot showing the protein amount of ATP6V1B2 in the cells treated with AllStars and ATP6V1B2 siRNAs.** β-actin actin was used as loading control.
(TIF)

Figure S3 **IAV infection in AllStars negative and ATP6V1B2 siRNA-treated cells.** The cells were fixed 8 h after viral inoculation, and processed for staining. In the infected cells, NP (green) is expressed. Nuclei are stained with Hoechst (blue).
(TIF)

Figure S4 **High-throughput microscopy images of the individual assays (EB, EE, EA, EF, EU, and EI assays), acquired with a 20× objective.**
(TIF)

Figure S5 **Sample images acquired by screening microscope.** (a) Uncoating (EU assay). Sample cells highlighted: 1. Uncoated cell with homogenous signal, 2. Uncoated cell containing several dots, 3. Non-uncoated cell without dots, 4. Non-uncoated cell with pronounced dots. (b) Nuclear import (EI assay). 1. and 2. E_I_ positive cells with and without dots, 3. E_I_ negative cell with dots. (c) Time-course plot of the EI assay using average number spots per cell as readout. The separation is not as clear and consistent between consecutive time-points compared to using machine learning-based separation (see Figure 3e). (d) Z’ factor and significance levels for using machine learning and simple spot detection to distinguish AllStars and ATP6V1B2 siRNA-treated cells.
(TIF)

Figure S6 **Comparison of different machine learning method performance for the EI assay.** (b) ROC plot for EI using *LogitBoost* method.
(TIF)

Figure S7 **Screenshot of the Advanced Cell Classifier program for the EU assay.**
(TIF)

Figure S8 **Binding of IAV on the cell membrane (EB assay) of AllStars negative and ATP6V1B2 siRNA-treated cells.**
(TIF)

Figure S9 **Validation of the EE, EA, EU, and EI assays with relevant positive controls.**
(TIF)

Table S1 **Summary of the virus amounts and the detection time-points of the EB, EE, EA, EF, EU, EI, and infection assays.**
(TIF)

Table S2 **Image analysis steps of each assay.**
(TIF)

Table S3 **Sequences of siRNAs targeting ATP6V1B2, ATP6AP2, ATP6V1A, CUL3, and CSE1L genes.**
(TIF)
