# Supplementary figures and images for: High-Content Analysis of Sequential Events during the Early Phase of Influenza A Virus Infection
Source: PLoS One. 2013 Jul 12;8(7):e68450. doi: 10.1371/journal.pone.0068450 (PMC3709902; doi:10.1371/journal.pone.0068450)

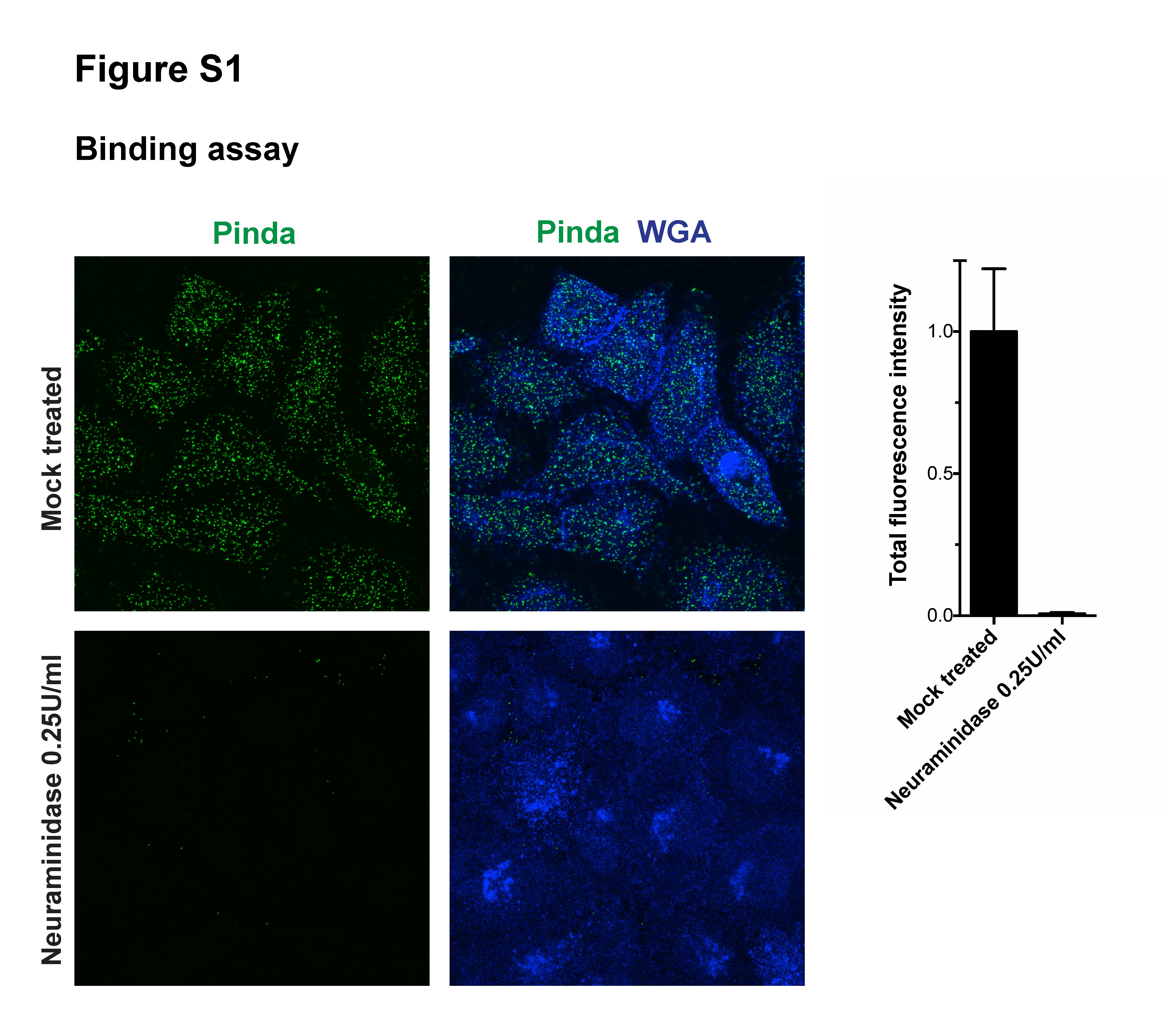

Supplement: Figure S1 — IAV binding in the neuraminidase and mock-treated cells. A549 cells were treated with 0.25 units/ml neuraminidase at 37°C for 4 h, followed by EB assay. Images were acquired with a confocal microscope. The HA of IAV was stained with Pinda antibody (green), and the cell membrane was stained with WGA (blue). (TIF) [file pone.0068450.s001.tif]

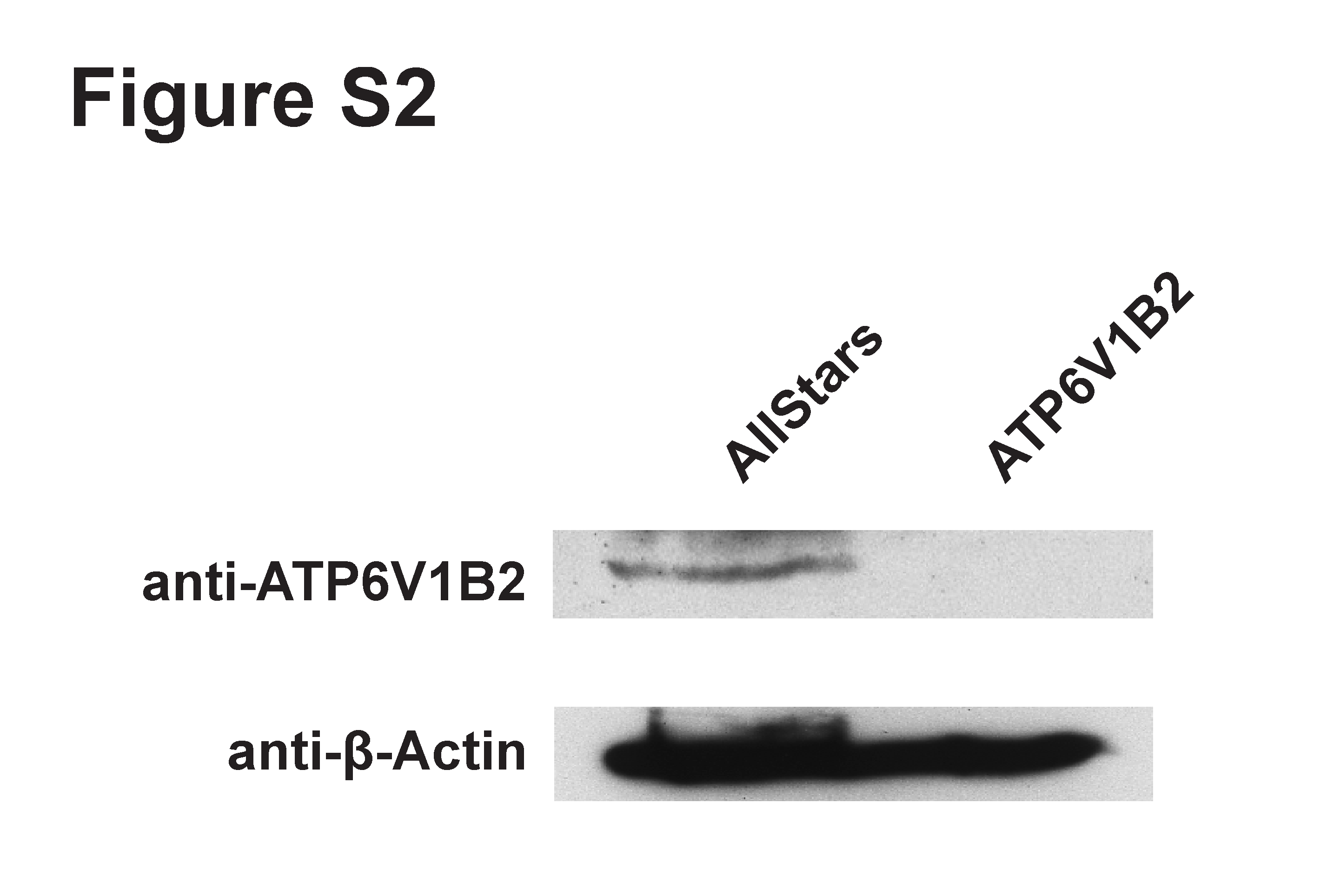

Supplement: Figure S2 — Western blot showing the protein amount of ATP6V1B2 in the cells treated with AllStars and ATP6V1B2 siRNAs. β-actin actin was used as loading control. (TIF) [file pone.0068450.s002.tif]

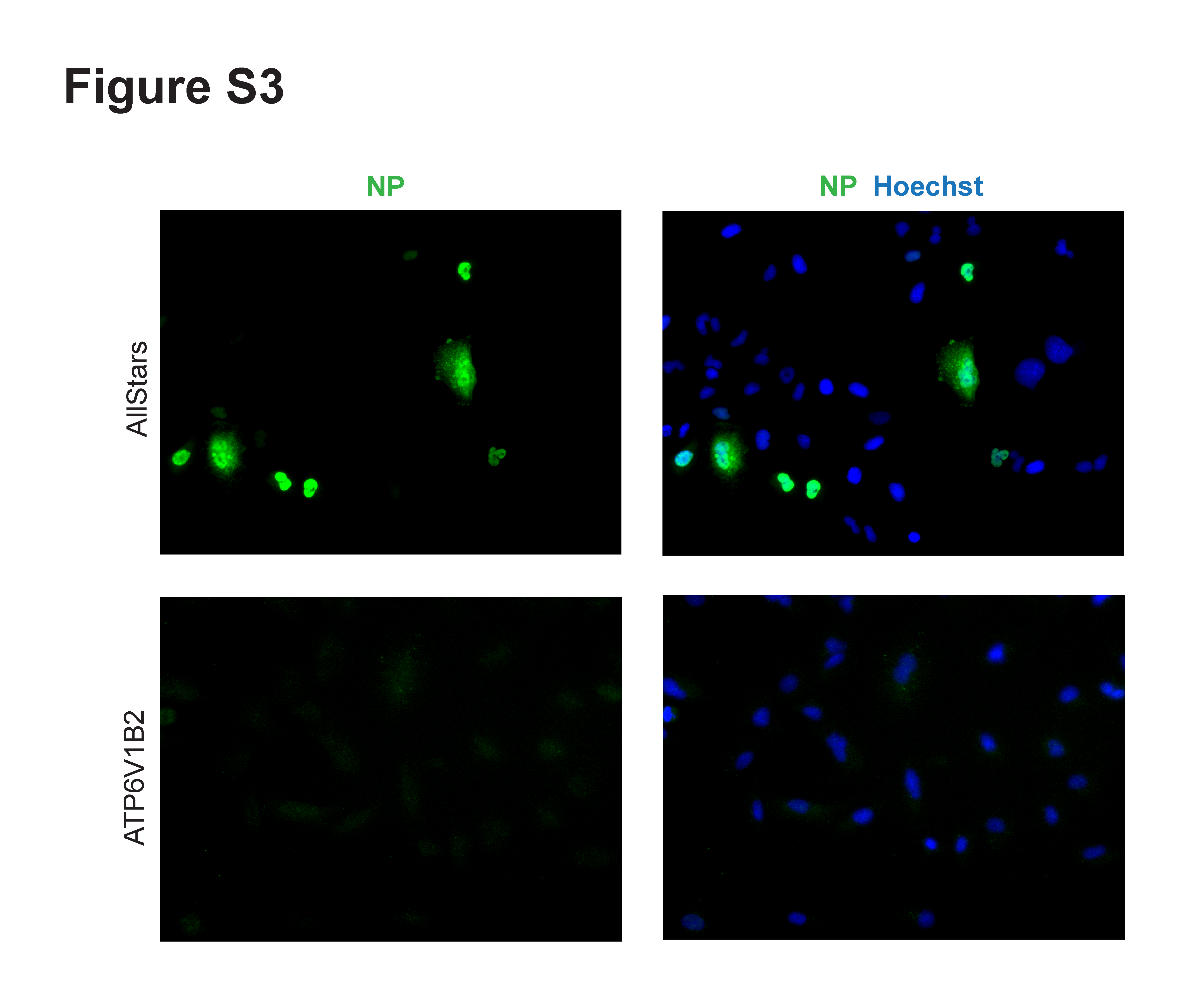

Supplement: Figure S3 — IAV infection in AllStars negative and ATP6V1B2 siRNA-treated cells. The cells were fixed 8 h after viral inoculation, and processed for staining. In the infected cells, NP (green) is expressed. Nuclei are stained with Hoechst (blue). (TIF) [file pone.0068450.s003.tif]

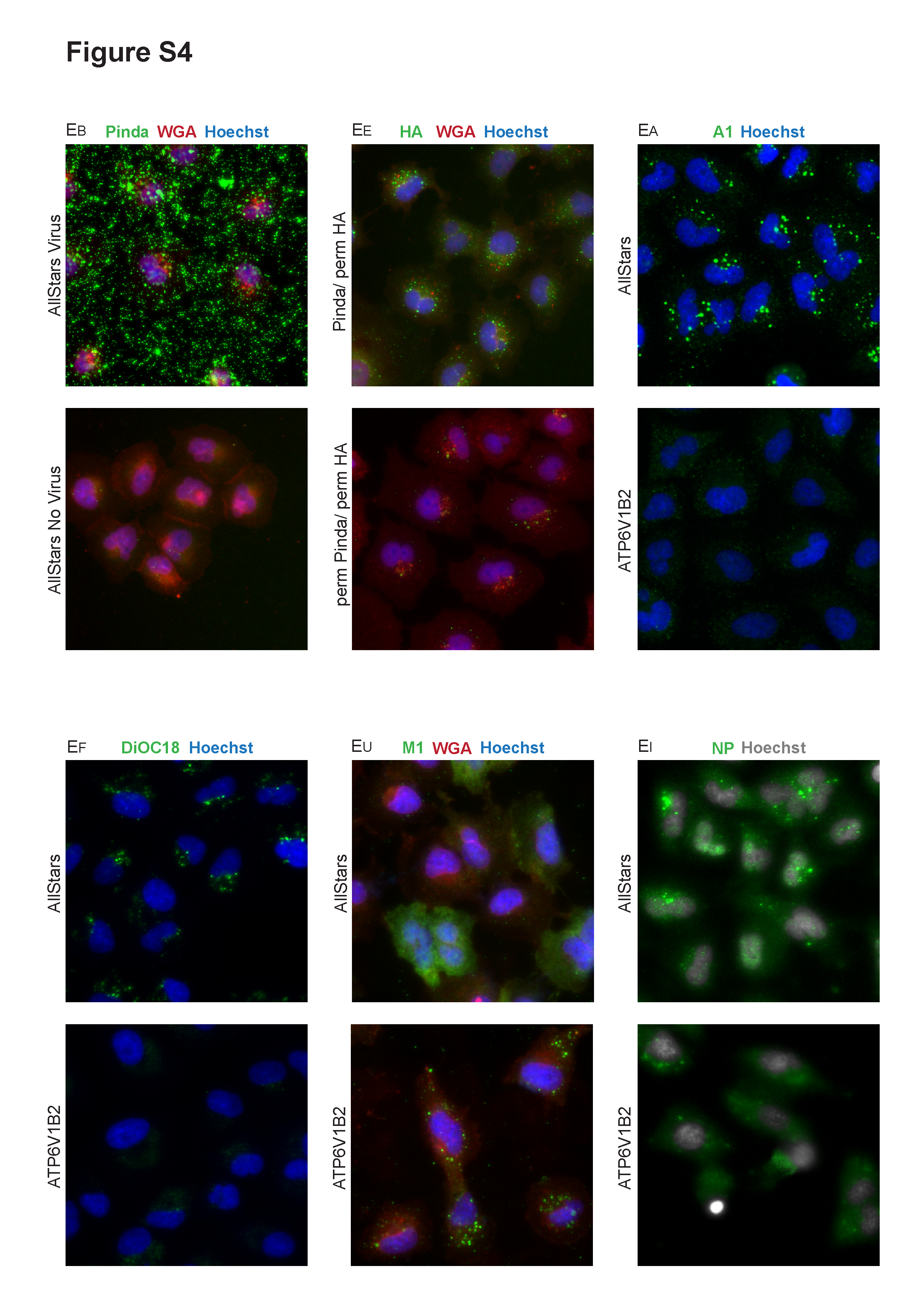

Supplement: Figure S4 — High-throughput microscopy images of the individual assays (EB, EE, EA, EF, EU, and EI assays), acquired with a 20× objective. (TIF) [file pone.0068450.s004.tif]

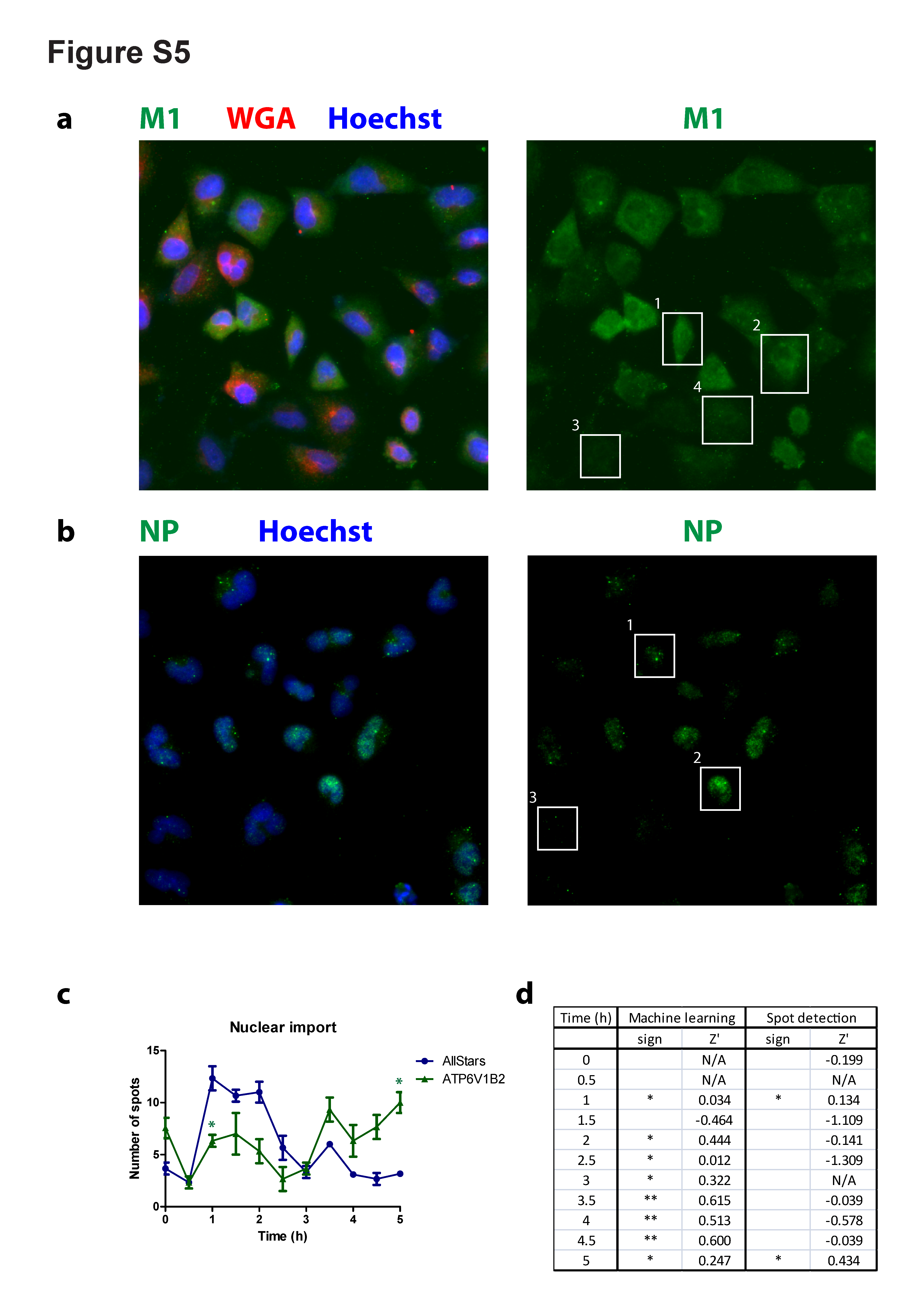

Supplement: Figure S5 — Sample images acquired by screening microscope. (a) Uncoating (EU assay). Sample cells highlighted: 1. Uncoated cell with homogenous signal, 2. Uncoated cell containing several dots, 3. Non-uncoated cell without dots, 4. Non-uncoated cell with pronounced dots. (b) Nuclear import (EI assay). 1. and 2. EI positive cells with and without dots, 3. EI negative cell with dots. (c) Time-course plot of the EI assay using average number spots per cell as readout. The separation is not as clear and consistent between consecutive time-points compared to using machine learning-based separation (see Figure 3e). (d) Z’ factor and significance levels for using machine learning and simple spot detection to distinguish AllStars and ATP6V1B2 siRNA-treated cells. (TIF) [file pone.0068450.s005.tif]

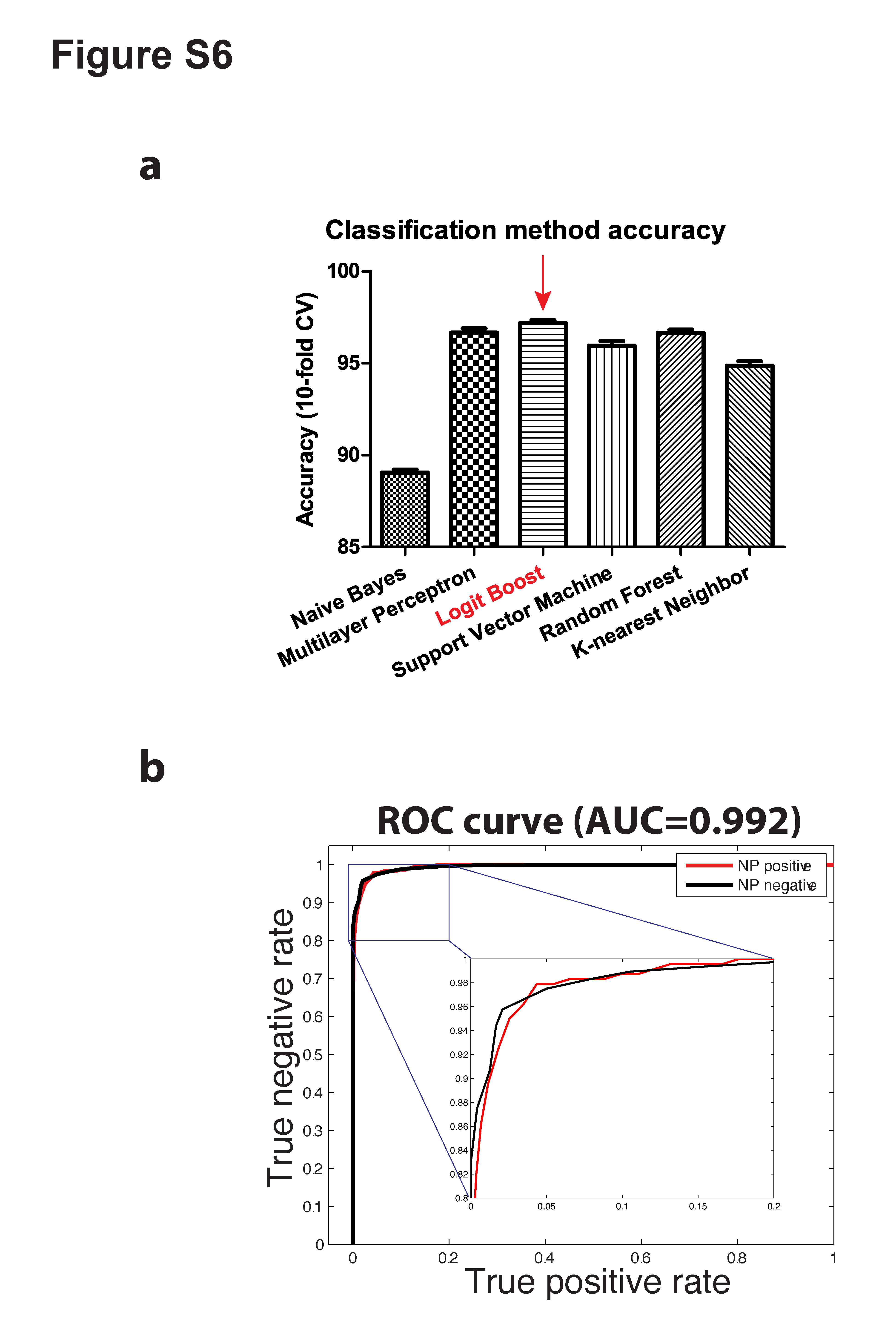

Supplement: Figure S6 — Comparison of different machine learning method performance for the EI assay. (b) ROC plot for EI using LogitBoost method. (TIF) [file pone.0068450.s006.tif]

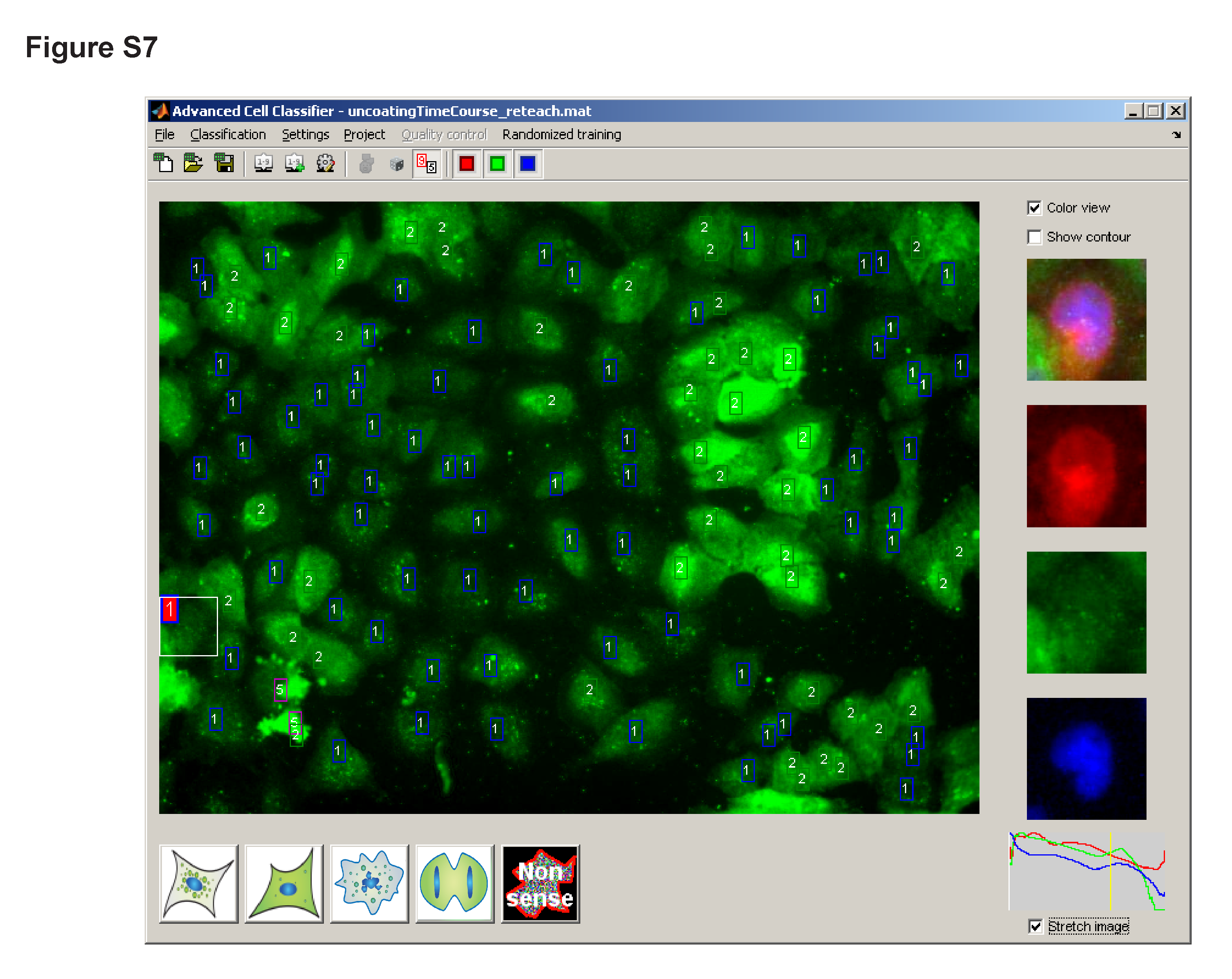

Supplement: Figure S7 — Screenshot of the Advanced Cell Classifier program for the EU assay. (TIF) [file pone.0068450.s007.tif]

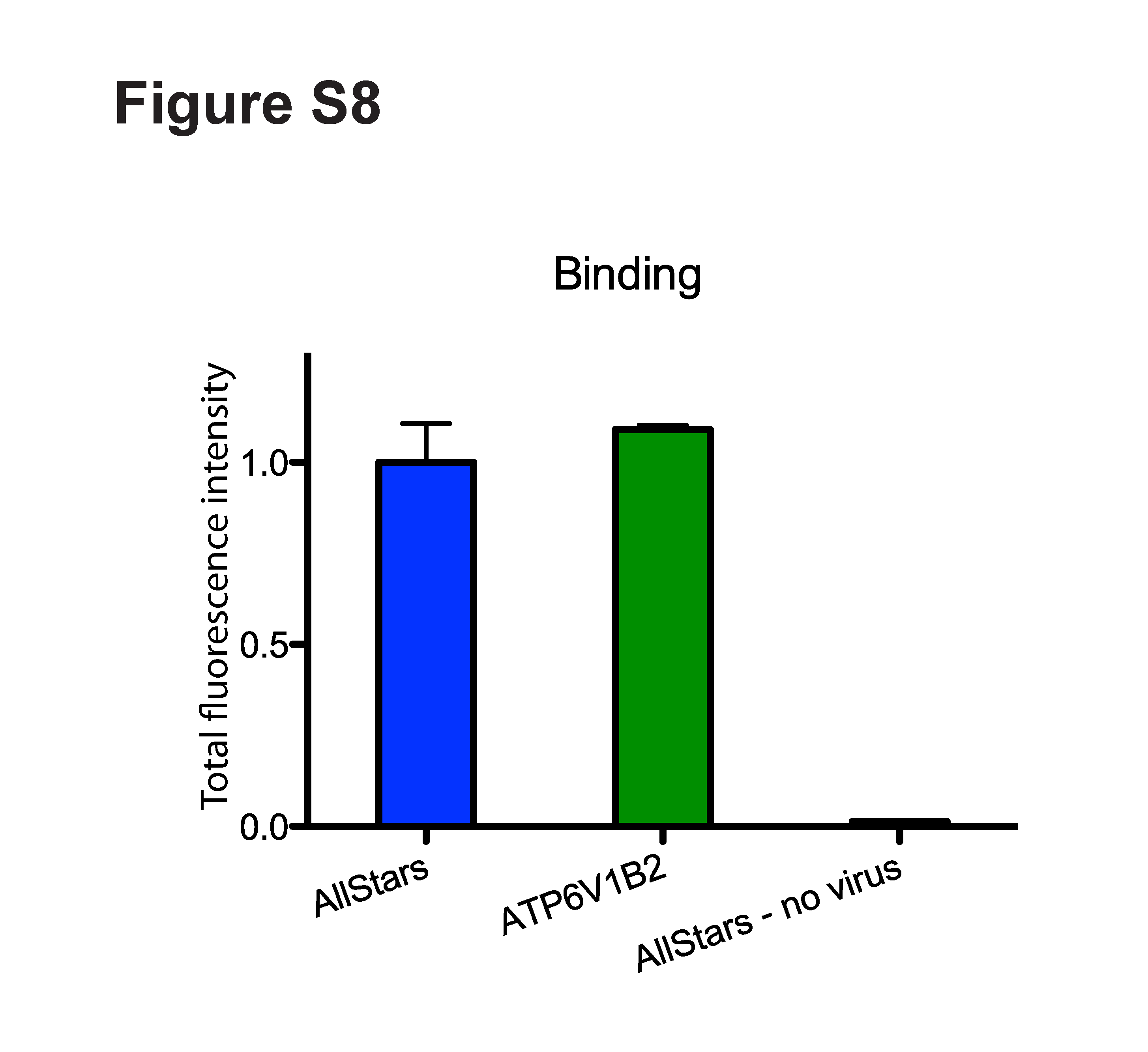

Supplement: Figure S8 — Binding of IAV on the cell membrane (EB assay) of AllStars negative and ATP6V1B2 siRNA-treated cells. (TIF) [file pone.0068450.s008.tif]

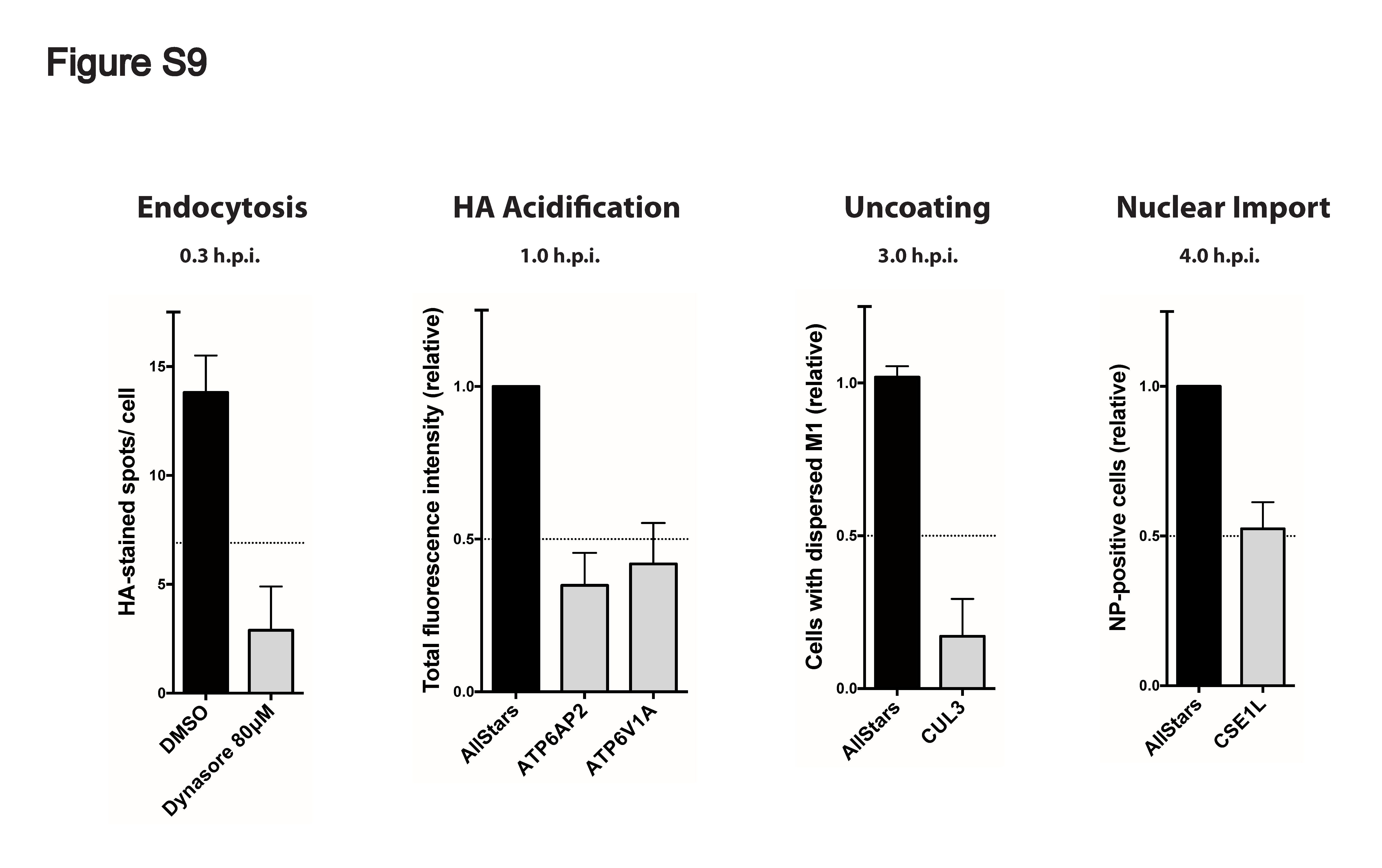

Supplement: Figure S9 — Validation of the EE, EA, EU, and EI assays with relevant positive controls. (TIF) [file pone.0068450.s009.tif]

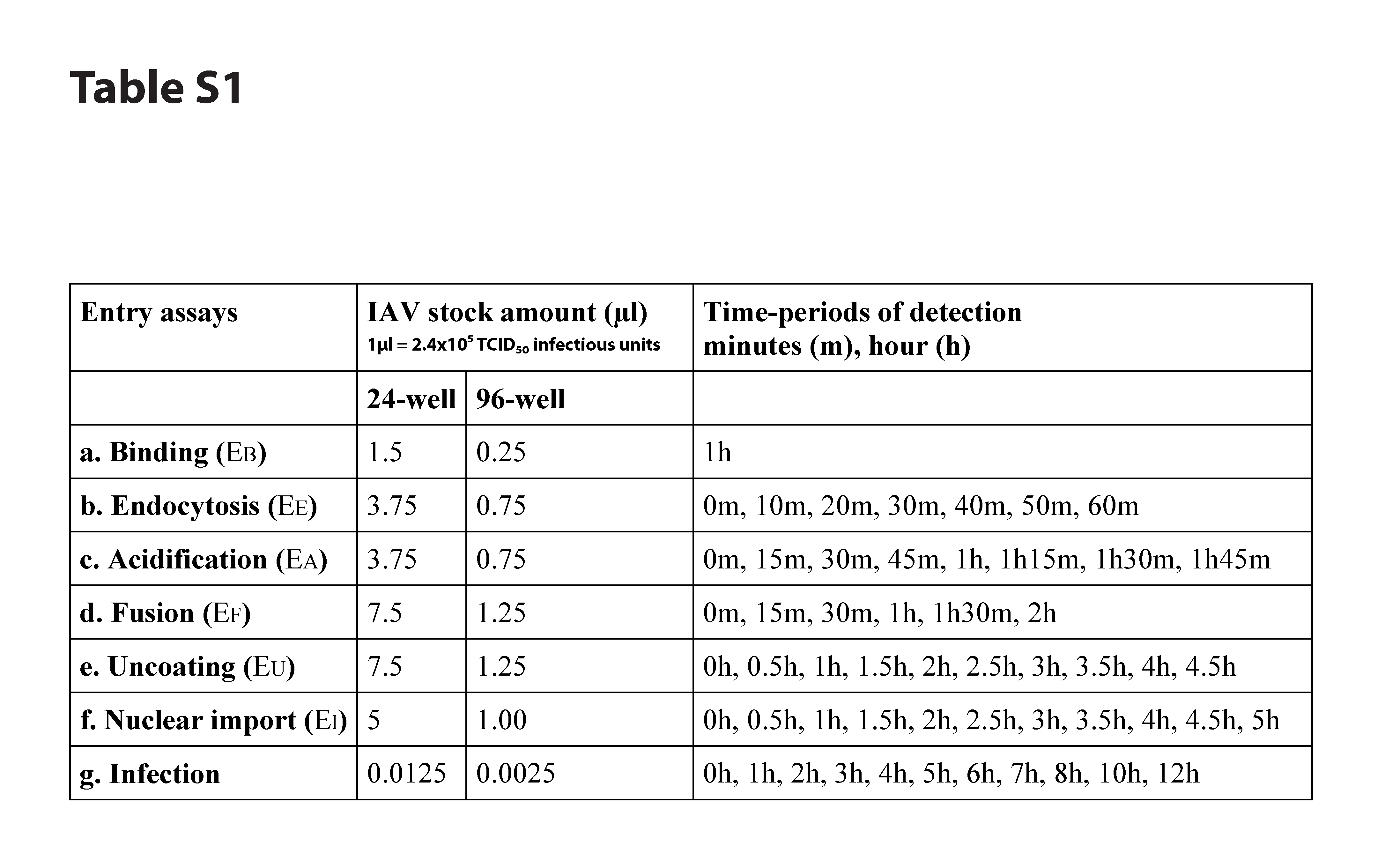

Supplement: Table S1 — Summary of the virus amounts and the detection time-points of the EB, EE, EA, EF, EU, EI, and infection assays. (TIF) [file pone.0068450.s010.tif]

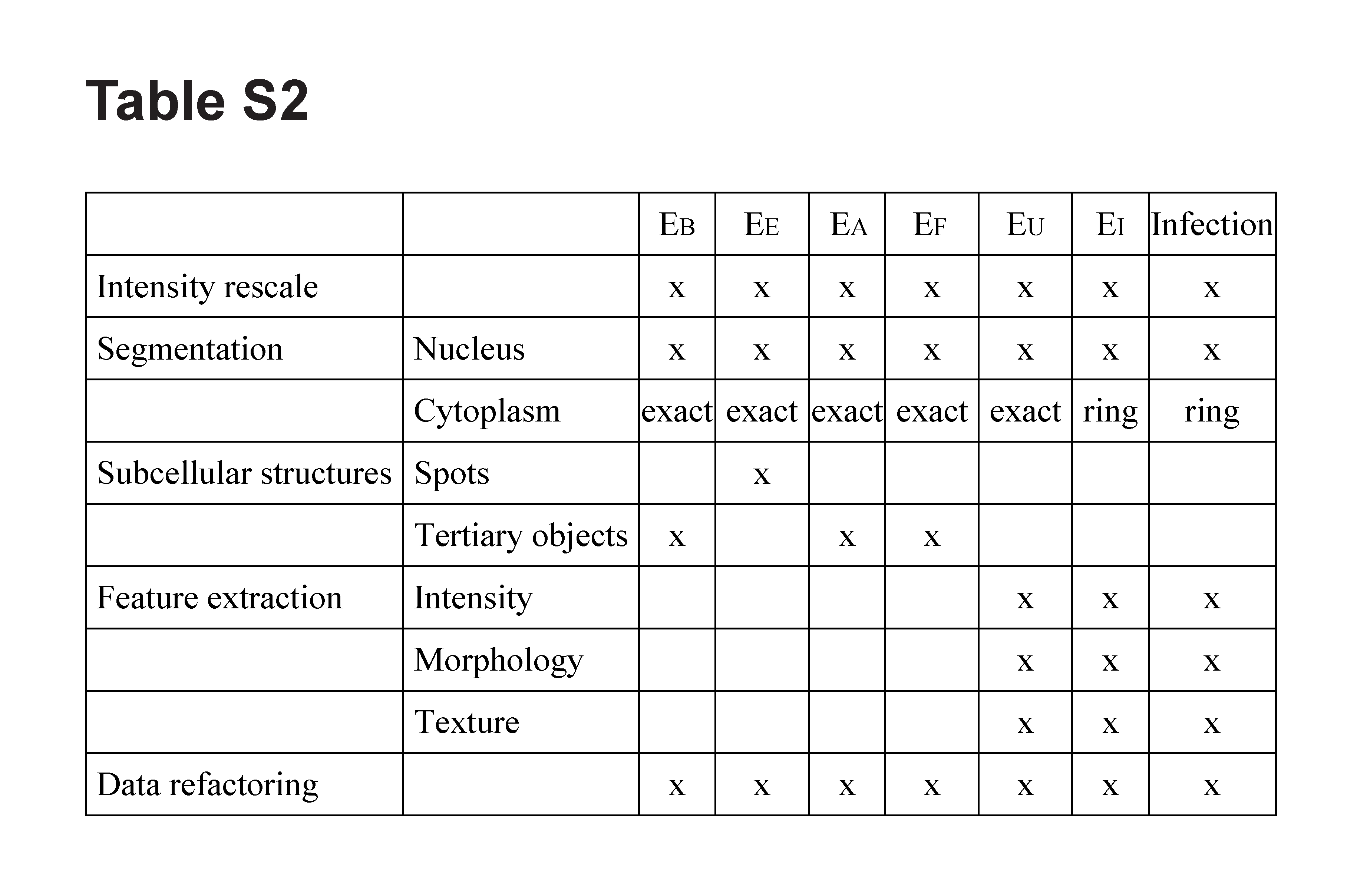

Supplement: Table S2 — Image analysis steps of each assay. (TIF) [file pone.0068450.s011.tif]

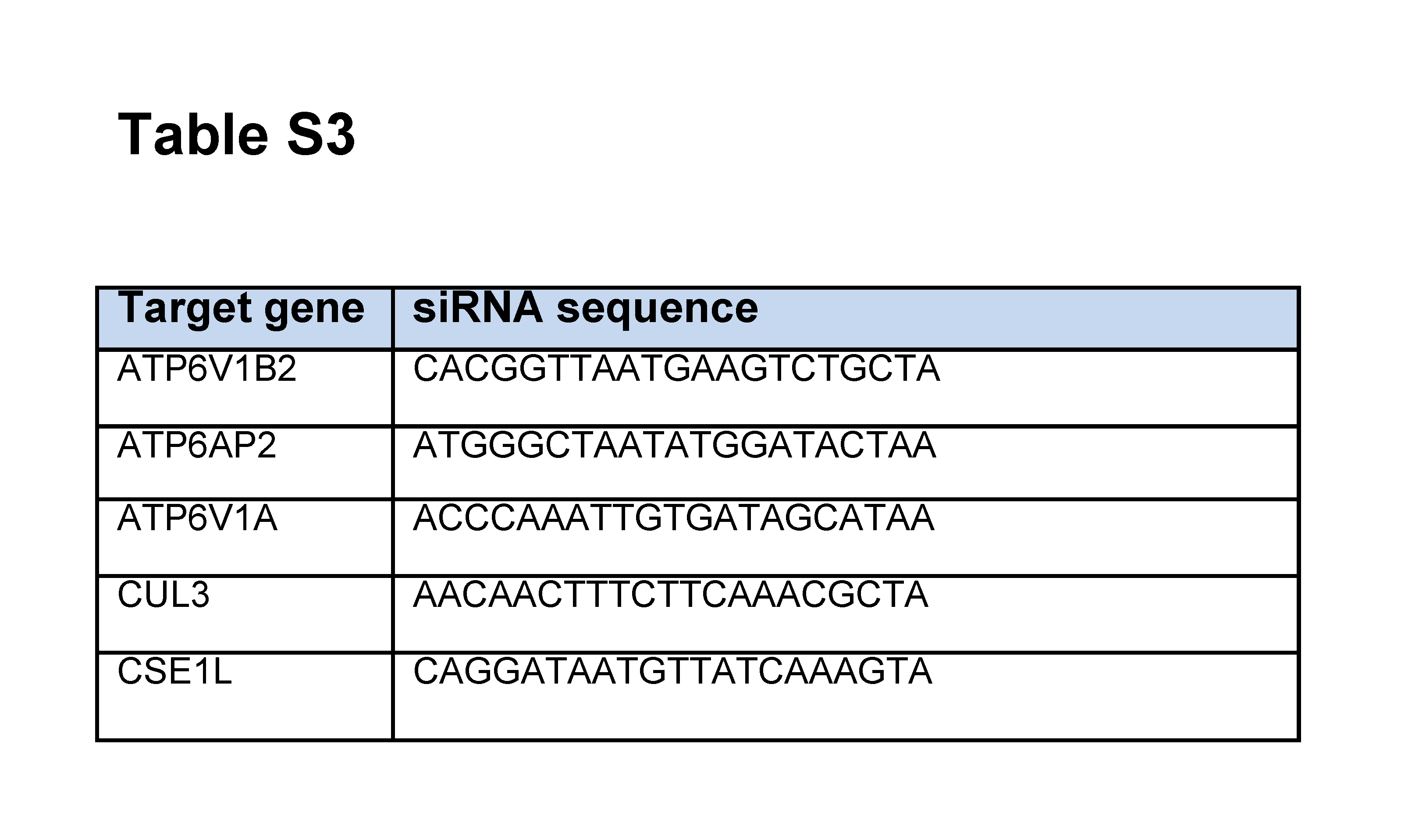

Supplement: Table S3 — Sequences of siRNAs targeting ATP6V1B2, ATP6AP2, ATP6V1A, CUL3, and CSE1L genes. (TIF) [file pone.0068450.s012.tif]
